# Whole-Genome Sequence of Ligilactobacillus faecis WILCCON 0062, Isolated from Feces of a Wild Boar (Sus scrofa)

**DOI:** 10.1128/mra.00376-23

**Published:** 2023-06-14

**Authors:** Yu Chyuan Heng, Shaktheeshwari Silvaraju, Sandra Kittelmann

**Affiliations:** a Wilmar International Ltd., WIL@NUS Corporate Laboratory, Centre for Translational Medicine, National University of Singapore, Singapore, Republic of Singapore; Wellesley College

## Abstract

We report the whole genome of a strain of Ligilactobacillus faecis. The complete circular chromosome and plasmid of strain WILCCON 0062 were obtained through a combination of short- and long-read sequencing and may be used to derive unprecedented insights into the genome-level phylogeny and functional capacities of Ligilactobacillus faecis.

## ANNOUNCEMENT

The species Ligilactobacillus faecis was first isolated from animal feces and described in 2013 (1). However, little is known about its physiology and metabolism, partly due to the lack of a genome sequence. Here, we present the whole-genome sequence of L. faecis WILCCON 0062, which was isolated from a fresh fecal sample from a wild boar (Sus scrofa). The sample was collected on the Island of Ubin, Singapore (1.4126°N, 103.9577°E), under permit NP_RP18-075/075a (National Parks Board, Singapore). An aliquot from the inner part of the sample, which was largely unexposed to oxygen, was serially diluted before being spread on MRS medium and incubated for 48 h at 37°C under anoxic conditions. Strain WILCCON 0062 was assigned to the species L. faecis based on 16S rRNA gene sequence similarity of 99.87% with respect to the type strain AFL 13-2 (GenBank accession number AB812750).

A single colony was grown in MRS broth at 37°C under anoxic conditions for 24 h, after which genomic DNA was extracted from the culture using the Maxwell 16 FFS nucleic acid extraction system (Promega). Short-read, paired-end sequencing was conducted using the Illumina HiSeq X Ten system (2 × 150-bp reads; NovogeneAIT Genomics) with a DNA library prepared using the NEBNext Ultra DNA library preparation kit and yielded 10,813,758 raw reads. Further quality trimming and filtering using Trimmomatic v0.39 (parameters: ILLUMINACLIP:TruSeq3-PE-2.fa:2:30:10 LEADING:3 TRAILING:3 SLIDINGWINDOW:4:15 MINLEN:36) (2) yielded 10,364,066 clean short reads. For long-read sequencing, the genomic DNA was sheared into fragments of 8,000 bp using a Covaris g-TUBE. The DNA library was prepared using the ligation sequencing kit SQK-LSK109, loaded onto a R9.4.1 flow cell, and sequenced using the MinION system (Oxford Nanopore Technologies [ONT]). Base calling and demultiplexing (strict mode) were conducted using Guppy v3.1.5 + 781ed57 (ONT). The 238,291 raw reads obtained were processed using Prowler (parameters: –windowsize 500 –minlen 1000 –trimode S –qscore 10 –clip LT –fragments F1) (3) to yield 160,923 clean long reads. The clean short and long reads were *de novo* assembled using Unicycler v0.5.0 (4) (with dependencies SPAdes v3.15.5 [5], Racon v1.3.3 [6], and BLAST+ v2.9.0+ [7]). The resulting hybrid assembly was evaluated using QUAST v5.0.2 (8) and checked for completeness and contamination using CheckM v1.1.3 (9).

The whole genome of strain WILCCON 0062 had a total size of 2,382,213 bp, a DNA G+C content of 39.48 mol%, and an average coverage of 947× (Table 1). It consisted of a circular chromosome of 2,379,201 bp and a circular plasmid of 3,012 bp. The plasmid contained the replication protein RepB (GenBank accession number WGN90609) and was estimated to have a copy number of 4.57 (based on relative read depth) (4). Annotation using NCBI Prokaryotic Genome Annotation Pipeline (PGAP) v6.5 (10) estimated the presence of 2,099 coding sequences (CDSs) (with 62 pseudogenes), 65 tRNAs (for 20 amino acids), 19 rRNAs (seven 5S rRNAs, six 16S rRNAs, and six 23S rRNAs), 1 transfer-messenger RNA (tmRNA), and 2 other noncoding RNAs (ncRNAs).

**TABLE 1:** Genome characteristics of Ligilactobacillus faecis WILCCON 0062

Parameter^*a*^	Finding
Strain	WILCCON 0062
BioProject accession no.	PRJNA956725
BioSample accession no.	SAMN34229426
Sequencing technologies	HiSeq X Ten (Illumina) and MinION (ONT)
No. of raw reads	
HiSeq X Ten	10,813,758
MinION	238,291
Total size of raw reads (bp)	
HiSeq X Ten	1,622,063,700
MinION	777,757,094
Raw read processing tool	
HiSeq X Ten	Trimmomatic v0.39
MinION	Prowler
SRA accession no. of clean reads	
HiSeq X Ten	SRR24201569
MinION	SRR24201568
No. of clean reads	
HiSeq X Ten	10,364,066
MinION	160,923
Total size of clean reads (bp)	
HiSeq X Ten	1,519,860,073
MinION	736,098,129
Clean read *N*_50_ (bp)	
HiSeq X Ten	150
MinION	5,812
Assembly tool	Unicycler v0.5.0 (hybrid assembly)
GenBank assembly accession no.	GCA_029889745
Whole genome size (bp)	2,382,213
Coverage (total bases/genome size) (×)	947
DNA G+C content (mol%)	39.48
Completeness (%)	98.69
Contamination (%)	1.05
Whole-genome content	1 chromosome and 1 plasmid
GenBank accession no.	
Chromosome	CP123639
Plasmid	CP123640
Size (bp)	
Chromosome	2,379,201
Plasmid	3,012
Circular	
Chromosome	Yes
Plasmid	Yes
No. of CDSs	
Chromosome	2,097
Plasmid	2
No. of CDSs with assigned KO no.	
Chromosome	1,117
Plasmid	0
No. of CAZymes	
Chromosome	62
Plasmid	0
No. of AMR/virulence genes	
Chromosome	0
Plasmid	0
No. of tRNAs	
Chromosome	65
Plasmid	0
No. of tmRNAs	
Chromosome	1
Plasmid	0
No. of other ncRNAs	
Chromosome	2
Plasmid	0
No. of rRNAs	
Chromosome	19
Plasmid	0
16S rRNA gene sequence GenBank accession no.	OQ842242
16S rRNA gene sequence length (bp)	1,561

a KEGG Orthology (KO) number assignment was performed using BlastKOALA v3.0 (11). Carbohydrate-active enzymes (CAZymes) and antimicrobial resistance (AMR) and virulence genes were identified using dbCAN3 (12) and ABRicate v1.0.1 (https://github.com/tseemann/abricate), respectively.

**FIG 1:**
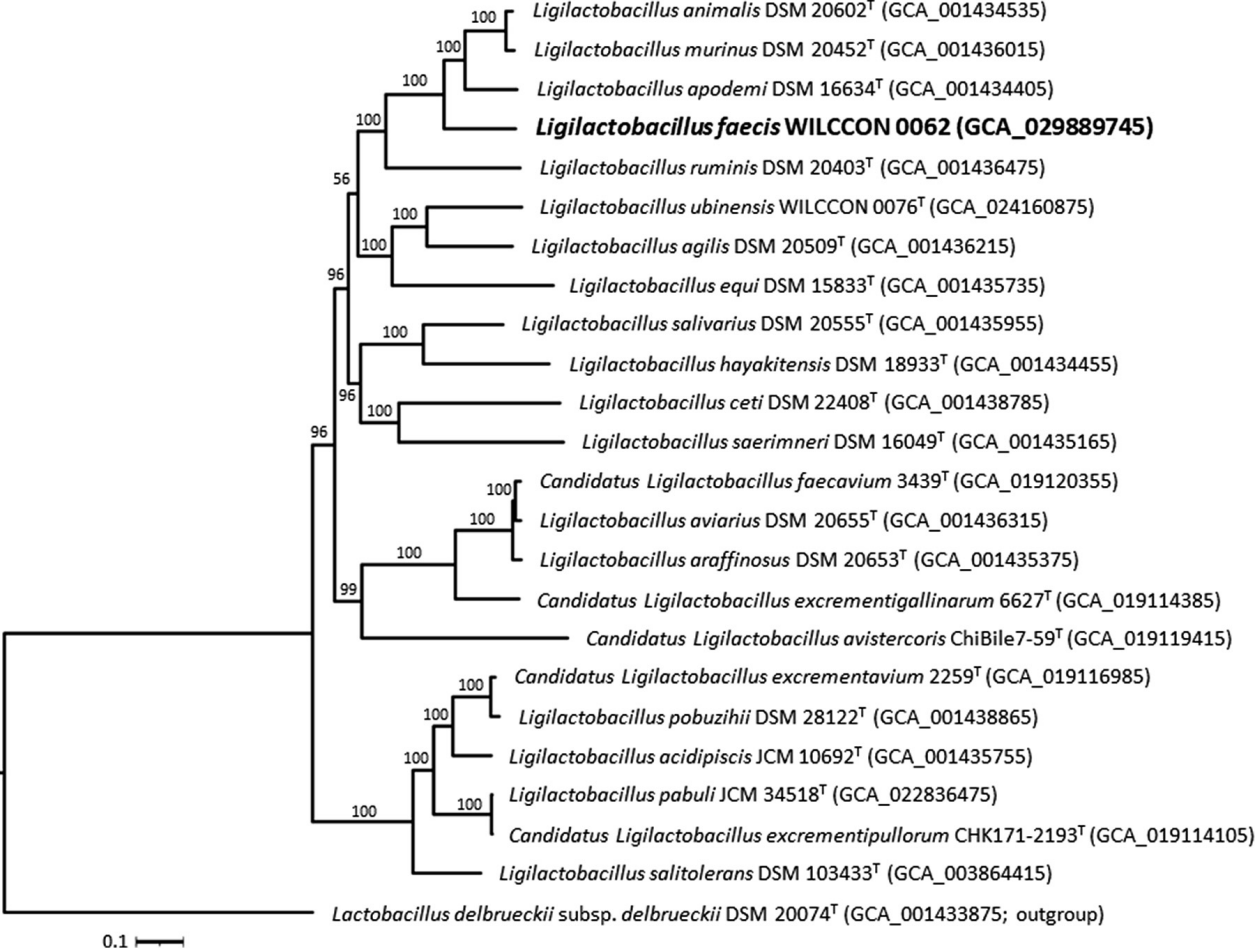
Phylogenomic tree showing the relationship between strain WILCCON 0062 (in bold) and closely related taxa of the genus *Ligilactobacillus*. The method for the reconstruction of the phylogenomic tree was described previously (13). Briefly, the protein sequences of 274 single-copy core genes of all included strains were concatenated and aligned using SCARAP v0.3.1 (14), and trimmed using trimAl v1.4 (15). The maximum-likelihood tree was inferred with IQ-TREE v2.1.2 (16) using the LG+F+I+G4 protein substitution model (optimal log likelihood of −1,622,429.979), rooted by midpoint rooting, and visualized using Interactive Tree Of Life (iTOL) v6.7.3 (17). Lactobacillus delbrueckii subsp. *delbrueckii* DSM 20074^T^ was used as an outgroup. Ultrafast bootstrap values based on 1,000 replications are indicated at the branching points. The scale bar denotes 0.1 substitutions per amino acid position. The GenBank assembly accession numbers are given in brackets.

The availability of a genome sequence of a strain of L. faecis allows not only ascertainment of its taxonomic position (Fig. 1) but also inference of information regarding its physiology and metabolism.

### Data availability.

The 16S rRNA gene sequence and the whole-genome assembly of Ligilactobacillus faecis WILCCON 0062 have been deposited in GenBank/ENA/DDBJ under accession numbers OQ842242 and GCA_029889745, respectively. The GenBank/ENA/DDBJ accession numbers for the circular chromosome and plasmid are CP123639 and CP123640, respectively. The Sequence Read Archive (SRA) accession numbers for the Illumina and ONT sequencing reads are SRR24201569 and SRR24201568, respectively. The associated BioProject and BioSample accession numbers are PRJNA956725 and SAMN34229426, respectively.
